# The colonic mucosa-associated microbiome in SIV infection: shift towards Bacteroidetes coincides with mucosal CD4^+^ T cell depletion and enterocyte damage

**DOI:** 10.1038/s41598-020-67843-4

**Published:** 2020-07-02

**Authors:** Kristina Allers, Christiane Stahl-Hennig, Tomas Fiedler, Daniel Wibberg, Jörg Hofmann, Désirée Kunkel, Verena Moos, Bernd Kreikemeyer, Jörn Kalinowski, Thomas Schneider

**Affiliations:** 1 Department of Gastroenterology, Infectious Diseases, and Rheumatology, Charité-Universitätsmedizin Berlin, Campus Benjamin Franklin, Hindenburgdamm 30, 12203 Berlin, Germany; 2 German Primate Center, 37077 Göttingen, Germany; 3 Institute of Medical Microbiology, Virology, and Hygiene, Rostock University Medical Centre, 18057 Rostock, Germany; 4 Center for Biotechnology (CeBiTec), Bielefeld University, 33615 Bielefeld, Germany; 5 Institute of Medical Virology, Charité-Universitätsmedizin Berlin, Campus Mitte, 10117 Berlin, Germany; 6 Present Address: Berlin-Brandenburg Center for Regenerative Therapies, Charité-Universitätsmedizin Berlin, Campus Virchow-Klinikum, 13353 Berlin, Germany

**Keywords:** Inflammation, Mucosal immunology, Infectious diseases, HIV infections, Pathogenesis, Infection, Inflammation, Gastroenterology, Gastrointestinal diseases

## Abstract

The intesinal microbiome is considered important in human immunodeficiency virus (HIV) pathogenesis and therefore represents a potential therapeutic target to improve the patients’ health status. Longitudinal alterations in the colonic mucosa-associated microbiome during simian immunodeficiency virus (SIV) infection were investigated using a 16S rRNA amplicon approach on the Illumina sequencing platform and bioinformatics analyses. Following SIV infection of six animals, no alterations in microbial composition were observed before the viral load peaked in the colon. At the time of acute mucosal SIV replication, the phylum Bacteroidetes including the Bacteroidia class as well as the phylum Firmicutes and its families Ruminococcaceae and Eubacteriaceae became more abundant. Enrichment of Bacteroidetes was maintained until the chronic phase of SIV infection. The shift towards Bacteroidetes in the mucosa-associated microbiome was associated with the extent of SIV infection-induced mucosal CD4^+^ T cell depletion and correlated with increasing rates of enterocyte damage. These observations suggest that Bacteroidetes strains increase during virus-induced mucosal immune destruction. As Bacteroidetes belong to the lipopolysaccharide- and short chain fatty acids-producing bacteria, their rapid enrichment may contribute to inflammatory tissue damage and metabolic alterations in SIV/HIV infection. These aspects should be considered in future studies on therapeutic interventions.

## Introduction

Mucosal inflammation and impairment of the intestinal barrier are among the earliest events in human immunodeficiency virus (HIV) infection^1–3^. Microbial translocations across the impaired intestinal barrier into circulation are thought to trigger systemic immune activation, which drives HIV disease progression^4,5^. Components of the intestinal microbiota produce factors that modulate epithelial growth and barrier function and regulate local immune responses^6^. Consequently, changes in the microbial composition have often been linked to inflammation and disease^7^. In HIV and simian immunodeficiency virus (SIV) infections, alterations in the intestinal microbiota are thought to contribute to pathogenesis^4,8–12^. However, the influence of the virus infection on the microbial composition remains poorly understood. Observational studies on HIV infection provide inconsistent results: in some lower diversity is described^10,13–15^, while in others no change or an overall higher diversity than in HIV-negative controls were observed^8,11,16,17^. Similarly, whereas several studies revealed a shift in the relative abundance from *Bacteroides* to *Prevotella* or *vice versa*^8,10,11,14,17^, others did not note a difference in these genera of the phylum Bacteroidetes^13,16^. Possible reasons for such contradictory results may include the cross-sectional study design, particularly in light of recent studies that suggest that the abundance of *Bacteroides* and *Prevotella* depends on sexual practice and lifestyle or nutrition rather than HIV infection^15,18,19^. Cross-sectional studies may not be suitable to provide information about cause-and-effect relationships, whereas longitudinal ones could be more valid for examining such relationships.

Data collected over a longer period of time are mainly derived from non-human primates (NHPs). However, all of these NHP studies used fecal samples for analysis, and tissue samples have never been analyzed longitudinally^20–23^. Nevertheless, with respect to immune pathogenesis, the more conserved mucosa-associated microbial compartment might be more important as it interacts with the mucosal immune system and resists the propulsion of water and debris through the intestine by attaching to the mucosa^24,25^.

Longitudinal studies about the impact of HIV/SIV infection on mucosal tissue-associated microbiome have not been performed yet. Moreover, data regarding the mucosa-associated microbiome in the early phase of infection are rare. Here we studied the composition of the colonic mucosa-associated microbiome within individual rhesus macaques before and during the course of SIVmac infection.

## Results

### Viral replication and CD4^+^ T cell depletion in the colonic mucosa of study animals

**Figure 1:**
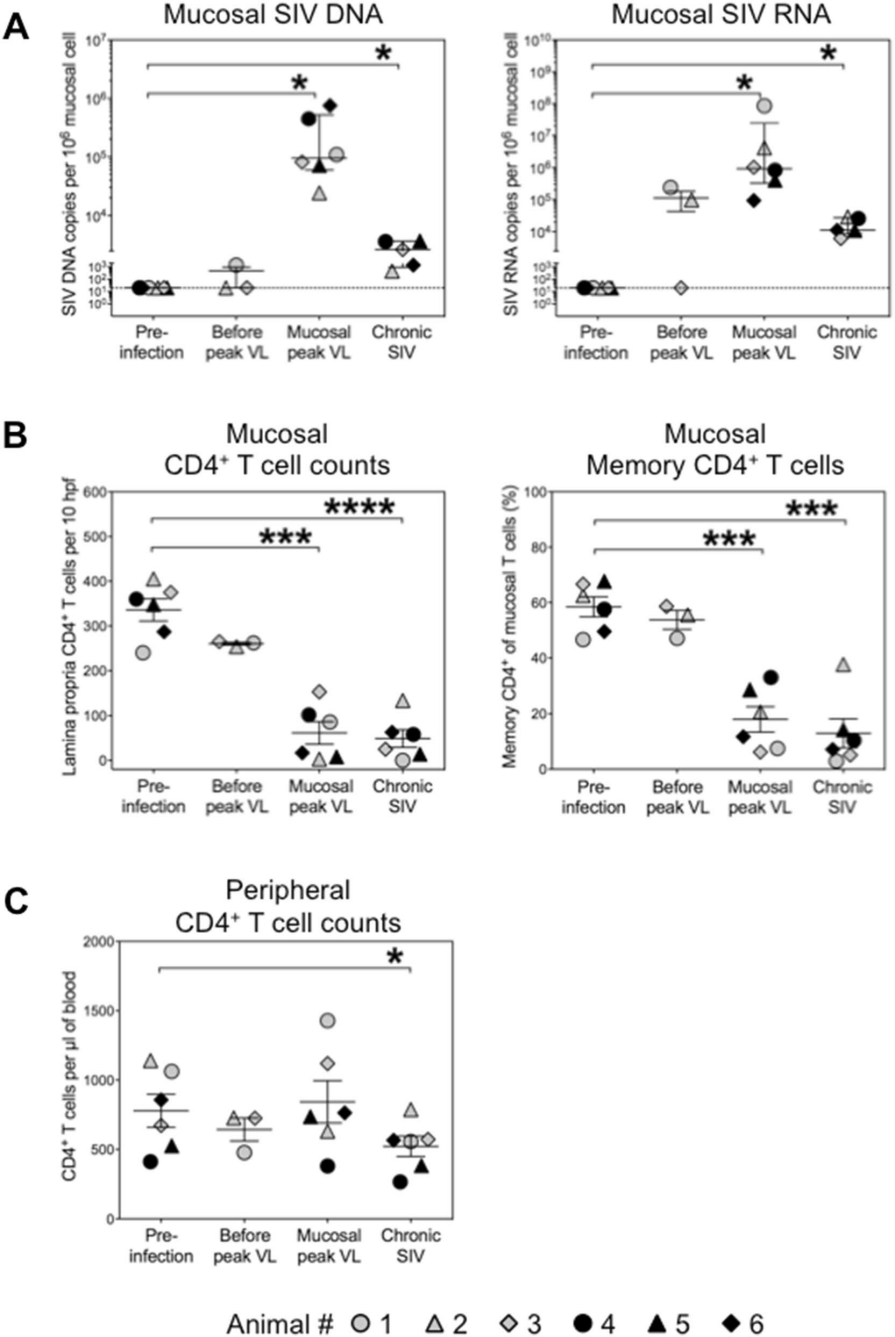
Mucosal SIV copy numbers and CD4^+^ T cells in the colon of SIV-infected animals. (**A**) Cell-associated SIV DNA and RNA copies were quantified longitudinally in colonic tissue before and after i.v. infection with SIVmac251. (**B**) CD4^+^ T cells in the colonic lamina propria were quantified by immunohistochemical analysis and the proportion CD45RA^-^ memory phenotype CD4^+^ cells within mucosal CD3^+^ T cells was determined by flow cytometry before and at different stages of SIV infection. (**C**) CD4^+^ T cell counts in the peripheral blood were measured in parallel. Mucosal peak viral load was observed on day 7 (black symbols) or on day 14 after infection (grey symbols). Day 7-samples of the three animals, in which mucosal viral load peaked on day 14, represent ‘before peak viral load’ data. Equivalent data samples were not available from animals in which mucosal viral load peaked already on day 7. The chronic phase was analyzed on day 49 after infection. Horizontal solid lines indicate mean values + /− standard deviations. The dashed horizontal line indicates the assay limit of detection. *P < 0.05, ***P < 0.001, ****P < 0.0001. *VL* viral load.

After cell-free and/or cell-associated virus transmission, variations in the time to mucosal viral peak were observed in the group of study animals, as described elsewhere^26^. In three of six animals, highest levels of SIV DNA and/or SIV RNA as well as depletion of mucosal CD4^+^ T cells were already observed on day 7 after infection, designated as time point of ‘mucosal peak viral load’ (Table 1; Fig. 1A, B). In the three other animals, SIV was either not detectable in the colonic mucosa at that time or present at only low levels, and therefore that time point was designated as ‘before peak viral load’ (Fig. 1A, B). Mucosal virus peak and loss of mucosal CD4^+^ T cells in these three animals were observed later, on day 14 after inoculation (‘mucosal peak viral load’) (Fig. 1A, B). During the chronic phase on day 49 post-infection (‘chronic SIV’), CD4^+^ T cells remained at low levels in five animals or were restored to some extent in one animal which presented with lowest mucosal SIV DNA levels during both infection phases analyzed (animal #2) (Fig. 1A, B). Mucosal SIV RNA and SIV DNA copy numbers during the course of infection are given in Table 1. CD4^+^ T cell counts in the peripheral blood are shown in Fig. 1C.

**Table 1:** Mucosal virus loads in the colon of SIV-infected animals.

No	Mucoal SIV DNA (copies/1,000 cells)^a^				Mucosal SIV RNA (copies/1,000 cells)^a^			
	d7	d14	d21	d49	d7	d14	d21	d49
1	1.4 < LOD < LOD **447** **71.8** **755**	**109** **24.3** **81.6** 35 30.5 29.7	22.1 4.4 8 7.7 35.9 12	ND 0.5 2.7 3.6 3.7 1.4	238 97.7 < LOD **831** **403** 93.8	**86,500** **4,160** **1,030** 7.8 246 **192**	1,810 369 173 19.9 186 47.8	ND 29 6.2 25.6 11.0 11.2
2								
3								
4								
5								
6								

*LOD* limit of detection, *ND* not determined

^a^Highest SIV DNA and SIV RNA loads in individuals during the course of infection are marked in bold

### Characterization of the colonic mucosa-associated microbiome in Chinese rhesus macaques housed in single cages

**Figure 2:**
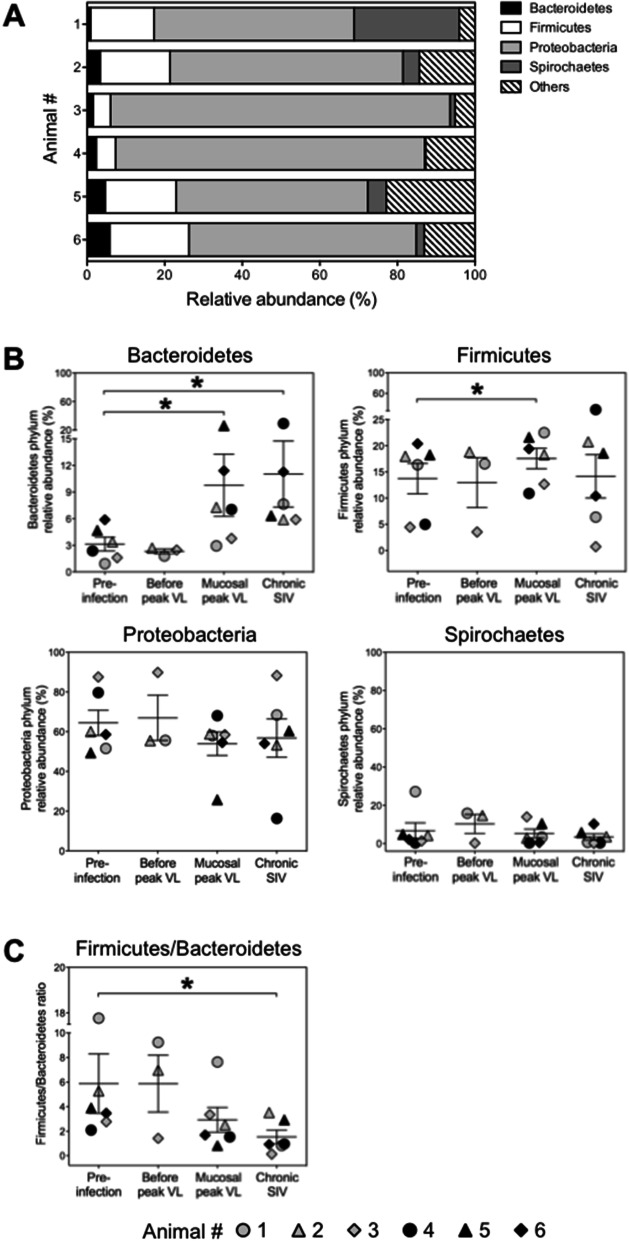
Composition of the colonic mucosa-associated microbiome at the phylum level before and after SIV infection. (**A**) Stacked bar representing the relative abundance of the top four most abundant phyla within colon biopsies in six animals before they were infected with SIV. Lower abundant bacteria (Actinobacteria, < 0.2%; Fusobacteria, < 0.2%; and Verrucomicrobia, < 0.5% in each animal) and bacteria that were not identified as belonging to any known phylum (mean 11%, range 3.8—22.6%) were termed ‘Others’. (**B**) and (**C**), Relative abundance of the four major phyla (**B**) and the ratio of Firmicutes to Bacteroidetes (**C**) before and at different stages of SIV infection. Values correspond to the frequency within the total bacteria detected within each animal. Mucosal peak viral load was observed on day 7 (black symbols) or day 14 after infection (grey symbols). The chronic phase was analyzed on day 49 after infection. Horizontal lines indicate mean values + /− standard deviations. *P < 0.05. VL, Viral load.

Colonic mucosa-associated microbial communities in single-caged rhesus macaques of Chinese origin were assessed in our six animals by 16S rRNA sequencing analysis before SIV infection. We identified four major microbial phyla representing more than 75% of the colonic mucosa-associated microbiome in each animal: Proteobacteria, Firmicutes, Bacteroidetes, and Spirochaetes (Fig. 2A). The most dominant bacterium in all six animals belonged to the Gram-negative genus *Helicobacter* (47.9–85.9%) of the phylum Proteobacteria. Analysis of full-length 16S rRNA showed closest matches to *Helicobacter macacae*. The next most abundant bacteria in five animals belonged to the Gram-positive families Lachnospiraceae (13.3–16.9%) or Ruminococaceae (0.9–1.9%) of the phylum Firmicutes*.* The second most abundant bacterial genus in one animal was *Brachyspira* (27.1%) from the Gram-negative phylum Spirochaetes.

### Impact of SIV infection on colonic mucosa-associated bacteria at the phylum level

To assess the influence of SIV infection on the microbial composition in colonic tissue, we first compared the abundances of the four major phyla at pre-infection to different time points after infection. Individual analysis of each of the four major phyla revealed notable alterations at the time of mucosal virus load peak. The relative abundances of Bacteroidetes and Firmicutes were increased 2- to 5.5-fold and 1.2- to 2.8-fold, respectively at that time (Fig. 2B). Bacteroidetes remained elevated during the chronic phase of SIV infection (Fig. 2B). The ratio of Firmicutes to Bacteroidetes decreased with disease progression, leading to a 1.5- to 22.5-fold reduction in the chronic phase (Fig. 2C), whereas the relative abundances of Proteobacteria and Spirochaetes did not change significantly during infection (Fig. 2B).

### SIV infection-induced alterations in the abundances of mucosa-associated bacteria below the phylum taxonomic level

**Figure 3:**
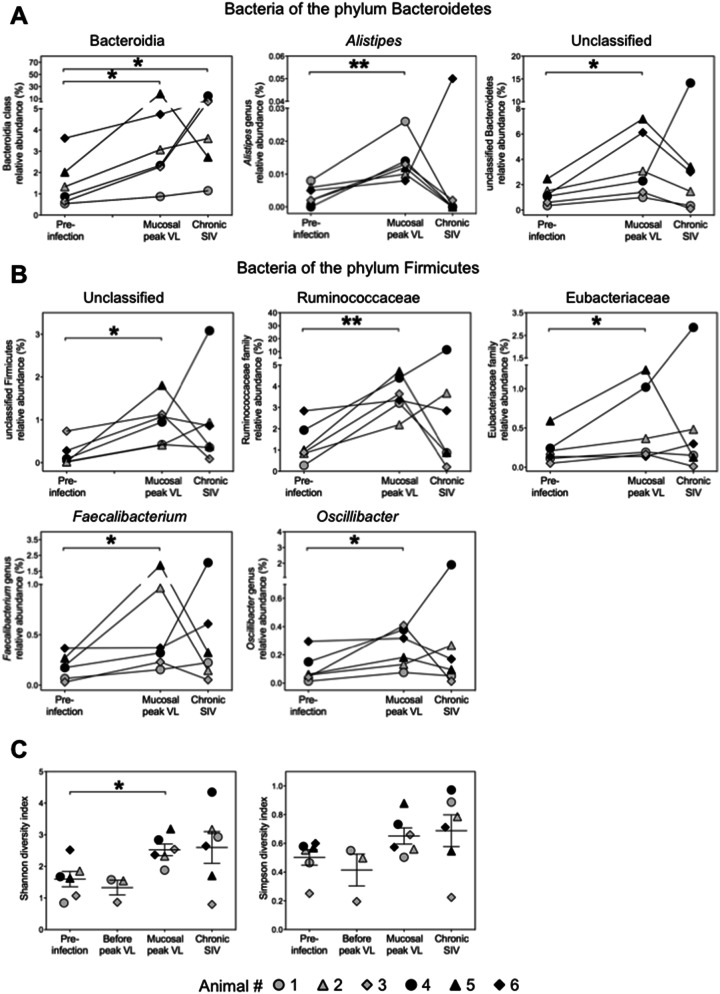
SIV infection-related shifts in the colonic mucosa-associated microbiome at phylum lower taxonomic levels and microbial diversity. (**A**) and (**B**), Relative abundance of bacteria among the phyla Bacteroidetes (**A**) and Firmicutes (**B**) before infection, at the time of mucosal viral load peak, and in the chronic phase of SIV infection. Values correspond to the frequency within the total bacteria detected within each animal. (**C**) Shannon diversity index and Simpson diversity index were calculated for each animal. Mucosal peak viral load was observed on day 7 (black symbols) or day 14 after infection (grey symbols). The chronic phase was analyzed on day 49 after infection. Horizontal lines indicate mean values + /− standard deviations. *P < 0.05, **P < 0.01. *VL* viral load.

Comparisons of the relative abundances of bacteria at the family level as well as the genus level revealed overall significant differences between pre-infection and the time of mucosal virus load peak (*P* < 0.0001 for both family and genus) and the chronic phase of SIV infection (*P* < 0.0001 for both family and genus). By contrast, before the colonic viral load peaked in the infected animals, there were no overall significant differences at the family or genus level in comparison with pre-infection levels (*P* = 0.98 and *P* = 0.41 for family and genus, respectively). Among the phylum Bacteroidetes, a shift to increased abundance was notable for the class Bacteroidia at the time of mucosal peak viral load (1.3- to ninefold) and during the chronic phase of SIV infection (1.5- to 17-fold) (Fig. 3A), while the class Flavobacteria did not change significantly (data not shown). At the genus level, *Alistipes*, which belongs to the family Rikenellaceae of the class Bacteroidia, was elevated in five animals (1.6- to 6.5-fold) at the time of mucosal virus peak load and became detectable for the first time in animal #4 (Fig. 3A). In addition, abundance of unclassified Bacteroidetes genera was raised 2-to fivefold at that time (Fig. 3A).

Among the Firmicutes phylum, no significant changes following SIV infection were observed in the abundance of the three major classes Clostridia, Bacilli, or Negativicutes (data not shown), but the abundance of unclassified Firmicutes genera was 1.5- to 90-fold increased at the time of mucosal peak viral load (Fig. 3B). At the family level, significant alterations in the abundance of bacteria were observed regarding increase in Ruminococcaceae and Eubacteriaceae families (1.2- to 11.5-fold and 1.7- to 4.2-fold, respectively) (Fig. 3B), whereas abundances of Lachnospiraceae and Veillonellaceae did not change (data not shown). At the genus level, significant rises were observed for *Faecalibacterium* and *Oscillibacter* (1.8- to 7.4-fold and 2.3- to 9.5-fold, respectively) from the family Ruminocaccacea at the time of mucosal viral load peak (Fig. 3B). No significant alterations during infection were observed among members of the phyla Proteobacteria and Spirochaetes (data not shown).

### Diversity in the colonic mucosa-associated microbiome before and after SIV infection

Taxonomic α-diversity was calculated by the Shannon index that accounts for richness and abundance distribution and the Simpson index, a measure of evenness. At the time of acute mucosal virus replication, the Shannon and the Simpson diversity index values were up to 2.4-fold and 2.6-fold higher as compared to those before infection in five of our six animals (Fig. 3C). The animal without increase at that time (animal #6) showed a comparatively high Shannon-diversity even before infection (Fig. 3C). At chronic stage of infection, diversity increased further in four animals and was comparable to pre-infection levels in two other animals (Fig. 3C).

### Relationships between alterations in the colonic mucosa-associated microbiome and immune parameters

**Figure 4:**
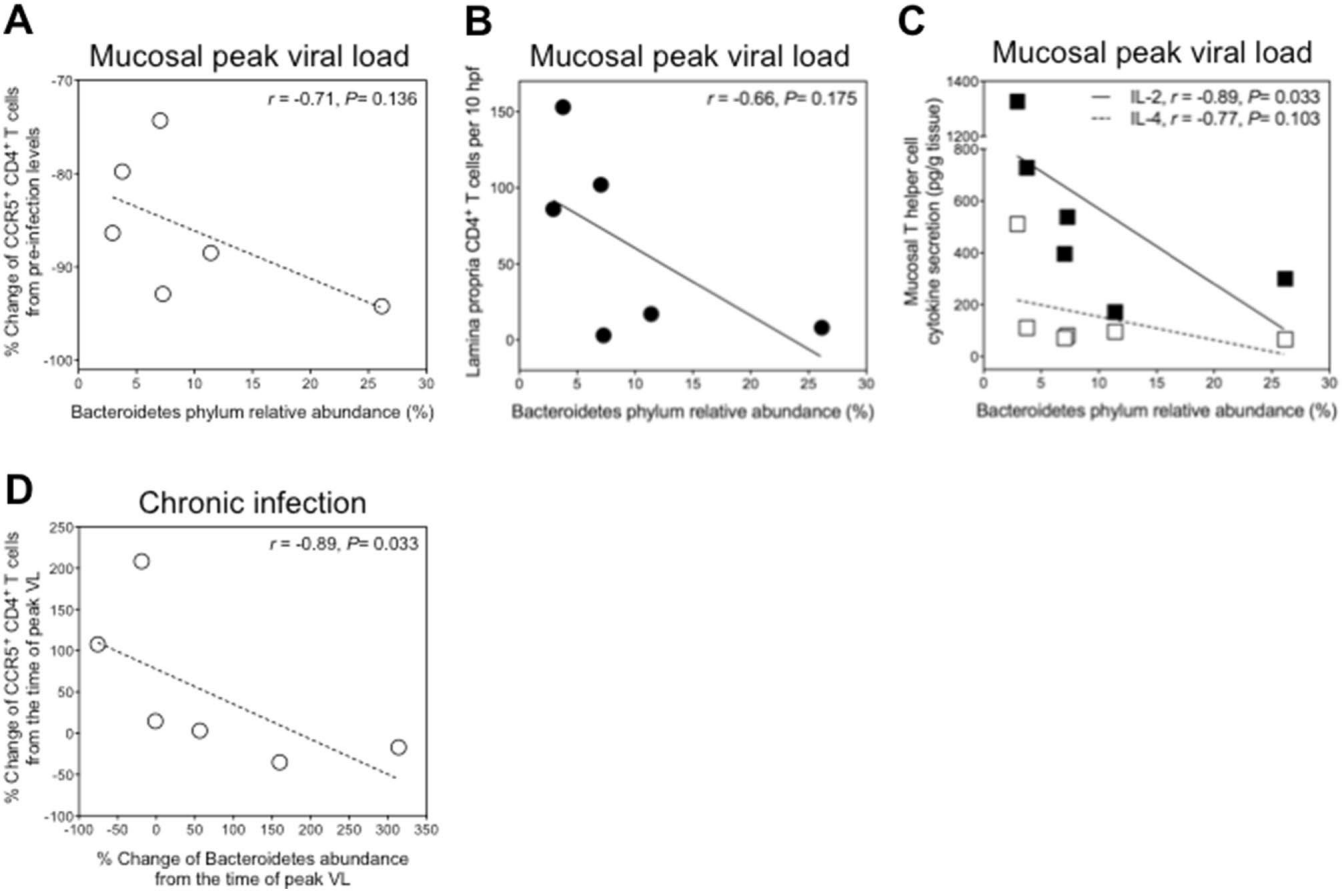
Associations of SIV infection-related shift towards Bacteroidetes with mucosal CD4^+^ T cell counts and cytokine secretion. Correlations of abundances of Bacteroidetes at the time mucosal peak viral load with (**A**) initial SIV infection-associated changes of frequencies of CCR5^+^ CD4^+^ T cells (determined by flow cytometric analysis) and (**B**) total CD4^+^ T cell numbers in the colonic mucosa (quantified by in situ immunostaining) and (**C**) with the levels of mucosal secretion of IL-2 (black symbols) and IL-4 (open symbols). Values, depicted as percent change in immune parameters, were calculated for each animal as change between pre-infection levels and those at the time of mucosal peak viral load. (**D**) Correlations between the changes in mucosal CCR5^+^ CD4^+^ T cell frequencies and the changes in abundances of Bacteroidetes during the chronic phase of SIV infection. Percent changes were calculated between levels at peak viremia and levels at the chronic stage of infection.

To further investigate whether the observed microbial shifts are related to SIV infection-induced immune alterations, we performed correlation analyses. For this, we performed longitudinal analysis of T cell subsets by flow cytometry and in situ quantitation and, in parallel, of the mucosal cytokine secretion. At pre-infection, i.e. under homeostatic conditions, there were no correlations of bacterial phyla with SIV target cells. At the time of mucosal peak viremia, under pathological conditions, the increased abundance of Bacteroidetes strongly correlated with the extent of mucosal C–C chemokin receptor type 5 (CCR5)^+^ CD4^+^ SIV target cell loss (Fig. 4A) and was negatively associated with total CD4^+^ T cell numbers (Fig. 4B) and CCR5^+^ CD4^+^ T cell frequencies (*r* = 0.6, *P* = 0.242) in the colonic mucosa. Although correlations did not reach statistical significance in this small group of animals, where the minimum *r*-value of 0.886 is required to yield *P*-values of ≤ 0.05, these trends indicate that the initial SIV infection-associated depletion of mucosal T helper cells might influence the proportion of Bacteroidetes within the mucosa-associated microbiome. Consistent with this, the abundance of Bacteroidetes at the time of mucosal peak viral load negatively correlated significantly with mucosal secretion of the T helper cell cytokine interleukin (IL)-2 and non-significantly with secretion of the type 2 cytokine IL-4 (Fig. 4C). In addition, after the time of mucosal peak viral load, there was a strong negative correlation between subsequent changes in the abundance of Bacteroidetes and the frequency of mucosal CCR5^+^ T cells (Fig. 4D). Negative correlation between changes of Bacteroidetes and total mucosal CD4^+^ T cell numbers was strong during the chronic phase of SIV infection (*r* = 0.66, *P* = 0.175) but did not reach statistical significance, probably due to the low sample size. Collectively, these observations indicate a relationship between the abundance of Bacteroidetes and the mucosal SIV-target cell depletion during the course of infection. Abundances of bacteria of the phylum Firmicutes, by contrast, did not correlate with mucosal immune parameters.

### Relationships of shifts in the mucosa-associated microbiome with enterocyte damage and microbial translocations

**Figure 5:**
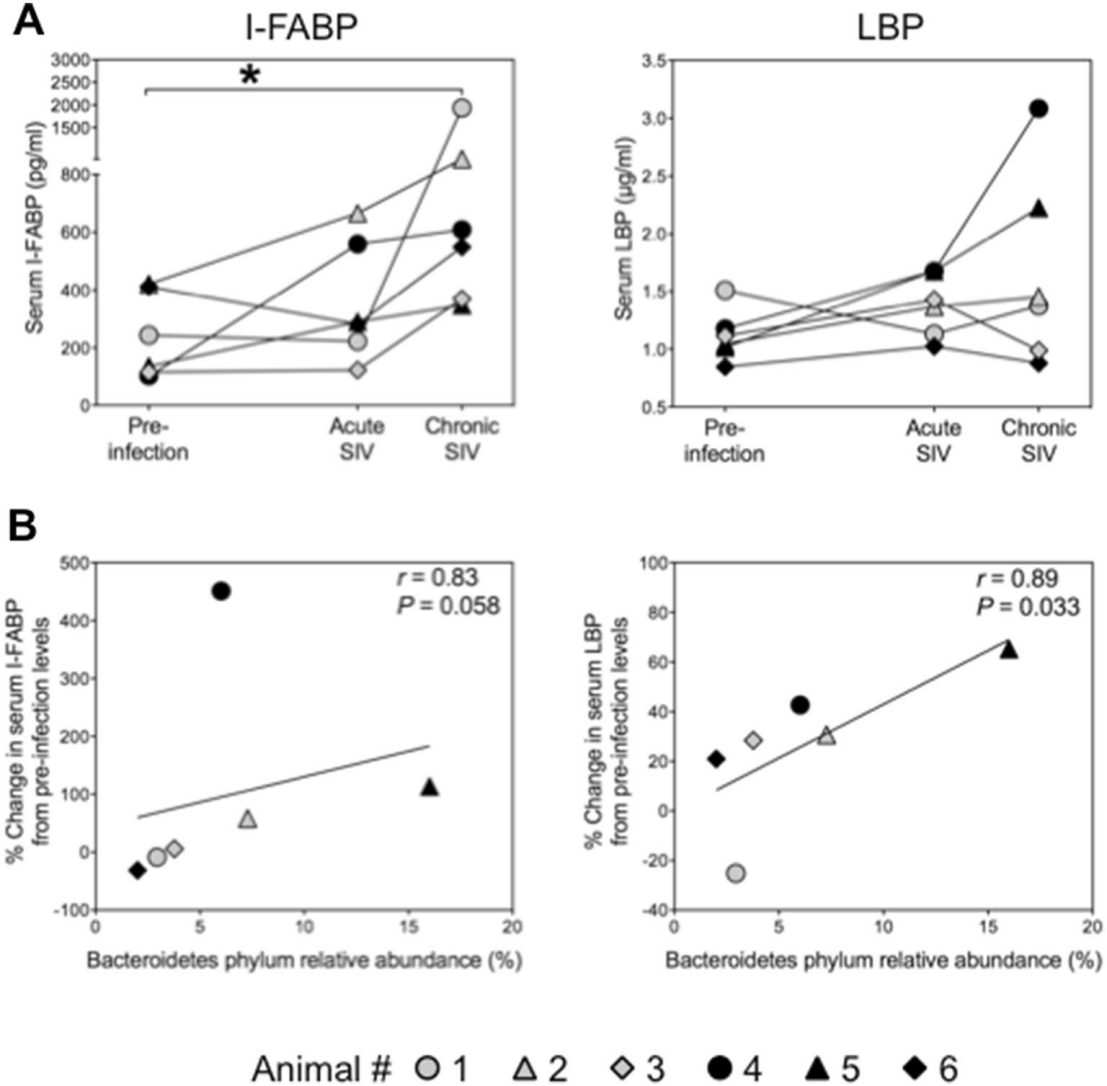
SIV infection-related impact on enterocyte damage in the colon. (**A**) Serum concentrations of I-FABP, a sensitive biomarker for enterocyte damage, and the microbial translocation biomarker LBP before infection and on days 14 and 49 after SIV infection (acute and chronic stage of infection). Mucosal peak viral load was observed on day 7 (black symbols) or day 14 after infection (grey symbols). The chronic phase was analyzed on day 49 after infection. (**B**) Correlations of abundances of Bacteroidetes with initial SIV infection-associated changes in I-FABP and LBP serum levels. Values, depicted as percent change in I-FABP or LBP, were calculated for each animal as change between pre-infection levels and those at the time of mucosal peak viral load. *P < 0.05.

To determine whether shifts in the microbiome coincide with the SIV infection-related mucosal barrier defect and subsequent microbial translocations into the systemic circulation, we quantified serum concentrations of intestinal fatty acid-binding protein (I-FABP), which is released into the bloodstream upon enterocyte damage and lipopolysaccharide (LPS)-binding protein (LBP), which is produced by the immune system in response to the gram-negative bacterial component LPS. As day 7-samples were not available for this analysis, samples collected on day 14 post-infection were tested for all animals (‘acute SIV infection’). Serum levels of I-FABP were increased in four animals with acute SIV infection (1.2- to 5.5-fold) and in all six animals during the chronic phase (1.3- to eightfold) (Fig. 5A), which indicates damage to the epithelial barrier that enhances intestinal permeability to microbial products. During the observation period, LBP levels increased in five animals (1.2- to 2.6-fold) after infection demonstrating the enhanced translocation of gram-negative bacterial components into systemic circulation (Fig. 5A). Early changes in I-FABP and LBP serum strongly correlated with abundances of Bacteroidetes (Fig. 5B) but not with those of Firmicutes or bacteria of other phyla (data not shown). Correlations of absolute levels of I-FABP and LBP with abundances of Bacteroidetes at the time of mucosal peak viral load (*r* = 0.6, *P* = 0.242 and *r* = 0.83, *P* = 0.058, respectively) were strong but did not reach statistical correlation due to sample size limitation.

In addition, there was a strong but non-significant correlation between the percent change in I-FABP levels from pre-infection to the time of chronic infection and concomitant percent change in the abundance of Bacteroidetes (*r* = 0.71, *P* = 0.136). These observations indicate that components or products of the phylum Bacteroidetes might affect the epithelial barrier integrity.

### Colonic mucosa-associated content of microbial products

**Figure 6:**
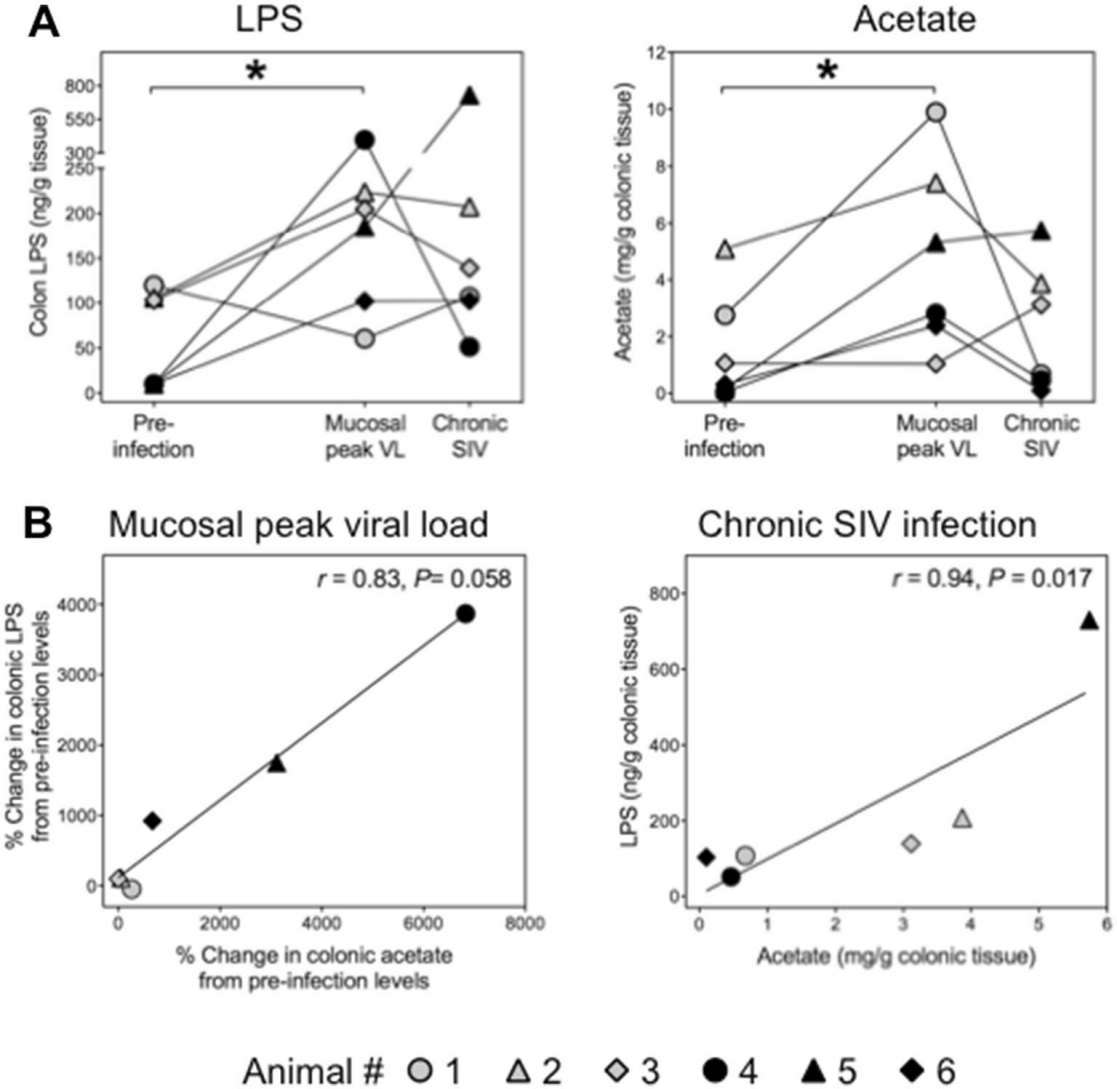
SIV infection-related impact on microbial components in the colonic mucosa. (**A**) Colonic mucosa-associated contents of LPS and acetate before and at different stages of SIV infection were quantified in tissue culture supernatants. Decay of bacteria and release of bacterial components into supernatant was achieved by antibiotics. Mucosal peak viral load was observed on day 7 (black symbols) or day 14 after infection (grey symbols). The chronic phase was analyzed on day 49 after infection. (**B**) Correlations between the initial changes of both bacterial components and between their levels at chronic stage of SIV infection. Values, depicted as percent change in LPS or acetate, were calculated for each animal as change between pre-infection levels and those at the time of mucosal peak viral load. *P < 0.05.

Bacterial components and fermentation end products have an impact on immune regulation and host metabolism. To investigate the influence of SIV infection on the release of such microbial products in the colon, we quantified the Gram-negative cell wall component LPS and the short chain fatty acids (SCFA) acetate and butyrate. At the time of acute mucosal virus replication, mucosa-associated contents of LPS and acetate were each increased in five out of six animals (2- to 40-fold and 1.5- to 69-fold) (Fig. 6A). In chronic infection, LPS levels remained higher as compared to those at pre-infection (Fig. 6A). The content of butyrate, by contrast, did not change significantly after SV infection (data not shown). There was a trend towards a strong correlation between the early changes in LPS and acetate levels and a very strong correlation between the levels of LPS and acetate in the chronic phase of SIV infection (Fig. 6B), which implies a causal or functional relation between mucosa-associated Gram-negative bacteria and acetate production in SIV infection. Together, these data indicate that SIV infection-associated shifts in the colonic microbiome may have metabolic effects.

## Discussion

In this longitudinal study of the microbiome, we have chosen biopsy tissue over stool samples for analysis to identify bacteria that are closely associated with the mucosal epithelial layer, and therefore are more likely to interact with mucosal cells and to translocate in the event of epithelial damage. Moreover, mucosal tissue samples were collected with only mild colonic cleansing preparation to reduce the potentially confounding effects of bowel preparation^27^.

One major finding is the shift towards Gram-negative Bacteroidetes in the colonic mucosa-adherent microbiome, including members of the Bacteroidia class, that was first observed at the time of mucosal virus peak, was maintained up to the chronic phase, and was associated with the extent of colonic CD4^+^ T cell depletion. Even though this association was not statistically significant at the time of mucosal peak viral load, probably due to the small number of animals, our observation is consistent with the previously observed trend toward a negative association between *Bacteroides* abundance and mucosal CD4^+^ T cells in the colon of HIV-infected persons^11^. Virus-induced mucosal immune defects might therefore play an essential role in shaping the tissue-associated microbial composition. Moreover, in spite of limitations due to small sample size, our data allow to speculate about a possible role of Bacteroidetes in the modulation of the epithelial barrier integrity, as their abundances were strongly associated with the rates of enterocyte damage. Interestingly, human diseases thought to be influenced by members of the phylum Bacteroidetes such as inflammatory HIV-related diseases bowel disease, colorectal cancers, and autoimmune disorders are all associated with impaired intestinal barrier integrity and enhanced microbial translocation^28–32^. In future studies, it would therefore be interesting to investigate whether specific components or products of Bacteroidetes may function as tissue disturbing factors, thereby increasing epithelial permeability and microbial translocations.

In addition, the abundance of Gram-positive Firmicutes and in particular bacteria of the Clostridia families Ruminococcaceae and Eubacteriaceae were increased at the time of acute mucosal virus replication. In contrast to the elevation of Bacteroidetes, these changes in microbial composition were not associated with mucosal immune parameters indicating that different mechanisms are involved in the SIV infection-related alteration of different bacterial phyla.

However, both bacterial phyla produce SCFAs from indigestible carbohydrates that reach the colon, with Firmicutes being the main butyrate producers and Bacteroidetes producing mainly acetate and propionate. In healthy condition, SCFAs are important energy sources for enterocytes, modulate intestinal processes such as electrolyte and water absorption and regulate immune responses^33^. Detrimental effects of tissue SCFAs including inhibition of effector functions and delay in wound repair have been described in infectious conditions and in the event of mucosal injury^34,35^. Progressive decrease in the ratio of Firmicutes to Bacteroidetes that we observed in this study may change SCFA production in the colon, thereby inducing metabolic alterations fostering disease progression^36^. Interestingly, a decrease in the Firmicutes/Bacteroidetes ratio has been shown to be associated with substantial weight loss^37^, a phenomenon that is often observed in HIV-infected patients during the course of infection and has often been considered as an unfavorable prognostic factor of survival^38,39^. We found in our SIV-infected animals an association of acetate production with increasing contents of LPS in colonic tissue, which indicates that Gram-negative bacteria can have metabolic effects in SIV infection. According to a previous study, acetate enhances HIV integration into the genome of host cells by histone modifications^40^. Consequently, high colonic acetate concentrations at the time of mucosal peak viral load might enhance viral integration and replication. Moreover, SCFAs have been shown to regulate colonic homeostasis by promoting regulatory T cell (Treg) differentiation^41^. Thus, alterations in the mucosa-associated microbiome composition might contribute to the previously described accumulation of colonic Treg in SIV infection^42^.

After infection, we additionally observed a trend toward a greater overall diversity of the tissue-associated microbiome with higher richness as measured by the Shannon diversity and evenness as measured by the Simpson diversity. This suggests that SIV infection-related changes in the colonic mucosa may facilitate the growth of diverse bacterial strains, including those of the phylum Bacteroidetes. By contrast to our tissue-associated findings, longitudinal studies of fecal microbiota showed reduced diversity or unchanged community composition during acute SIV infection^20,21^. Thus, the effects of SIV infection on the mucosa-associated microflora may differ from those on fecal microflora. Differences between the mucosa-associated and the luminal microbiome have previously been shown in healthy adults^25,43,44^.

The dominant genus within the mucosa-associated microbiome in the colon of our Chinese rhesus macaques was *Helicobacter* of the phylum Proteobacteria, which is in agreement with a previous study on healthy rhesus macaques^45^. In humans, by contrast, organisms from the phylum Proteobacteria are a minor population or absent in the colon^11,27,43^. High abundance of mucosa-adherent *Helicobacter* at this site may therefore be a distinctive feature of housed rhesus macaques. Colonization may result from early microbial exposures or through cross-infections via fomites by the fecal–oral route of transmission. Interestingly, persistent colonization of *Helicobacter* species has been demonstrated in the intestine of single-caged rhesus macaques^46,47^ but not in free living gorillas, chimpanzees, and bonobos^48^, which indicates that the host-environment shapes distribution.

In summary, our results suggest that alterations in the colonic mucosa-associated microbiome are induced at the time of acute mucosal SIV replication and are partly associated with mucosal CD4^+^ T cell depletion. Alterations in colonic microbial composition were dominated by an increase of LPS-containing and SCFA-producing bacteria of the Gram-negative phylum Bacteroidetes, which may contribute to inflammatory and metabolic disease in SIV/HIV infection. Moreover, association of the compositional shift towards Bacteroidetes with increasing rates of enterocyte damage suggests that mucosa-associated members of this phylum might contribute to the epithelial barrier defect and microbial translocations in SIV infection. As these pathological events are leading to HIV-related diseases in untreated and treated patients, that aspect could be important for future studies on therapeutic interventions.

## Methods

### Experimental SIV infection

Purpose-bred adult rhesus macaques (*Macaca mulatta*) of Chinese origin were housed at the German Primate Center (DPZ) according to the German Animal Welfare Act which complies with the European Union guidelines on the use of NHPs for biomedical research and the Weatherall report. The study was approved by the Lower Saxony State Office for Consumer Protection and Food Safety and performed with the project license 509.42502/08-04.03. Six animals were infected intravenously (i.v.) with cell-free SIVmac251. Three of those animals were additionally inoculated i.v. with cell-associated SIVmac251, as described elsewhere^26^. Transmission of cell-associated virus in these animals accelerated the initial virus dissemination^26^. Colonic biopsies were collected seven days before and at several time points after infection under combined ketamine, xylazine and atropine anaesthesia as previously described^26,42^. One day before colonoscopy, animals received Bisacodyl orally in water (1.2 – 2.7 mg/kg; Dulcolax, Sanofi-Aventis Germany) to stimulate gut motility. None of the animals developed acquired immunodeficiency syndrome-like disease during the observation period of 49 days. Plasma viral loads are given elsewhere^26^.

### Quantitation of viral DNA and RNA in mucosal tissue

DNA and RNA were extracted from colonic biopsy specimens with the use of the Allprep DNA/RNA Mini kit (Qiagen) according to the manufacturer’s protocol. Viral DNA or viral RNA were quantified by Taqman based real-time PCR (ABI-Prism 7500 Real-Time PCR system, Applied Biosystems) as described before^26,42^.

### Preparation and sequencing of 16S rRNA amplicon libraries

For phylogenetic analysis the V4 region of 16S rRNA genes was amplified from colon DNA using the Illumina overhang-adapter-sequence coupled primers 515F (5′GTGCCAGCMGCCGCGGTAA3′) and 806R (5′GGACTACHVG GGTWTCTAAT3′)^49^ as described in the Illumina “16S Metagenomic Sequencing Library Preparation” instructions with 30 amplification cycles. Amplicon size (390 bp including adaptor sequences) and quality was checked for each sample by use of Agilent Bioanalyzer DNA1000 chips. The dual-indexing PCR was performed as described in the above-mentioned manual. Quality of the final libraries was again checked on Agilent Bioanalyzer DNA1000 chips. Each individual library was adjusted to a DNA concentration of 4 nM. DNA concentration was measured applying Qubit assay kits (Invitrogen). The libraries were pooled, spiked with a PhiX control as recommended in the Illumina “16S Metagenomic Sequencing Library Preparation” protocol and diluted to a DNA concentration of 3 pM. Paired-end sequencing was performed on an Illumina MiSeq machine using a MiSeq Reagent Kit v3 for 600 cycles following manufacturer’s instructions. The resulting fastq raw data files were used for data analysis. These raw data are available in EMBL-EBI SRA under the Bioproject ID PRJEB31033.

### High-throughput 16S rRNA gene amplicon analyses

Adapter and primer trimming of amplicon raw reads were performed through an inhouse pipeline^50^. Based on a pipeline including FLASH v.1.2.11^51^, USEARCH v.10^52^, UPARSE^53^, and the Ribosomal Database Project (RDP) classifier v.2.9^54^, all amplicons datasets were processed as recently described^55–58^ (For details see: https://www.drive5.com/usearch/manual10/ex_miseq.html). In brief, all sequences that were not merged by FLASH (default settings with modifications [M = 300]), sequences with > 1 N (ambiguous base) in the sequence read and expected errors > 0.5 were discarded. Filtered datasets were processed, operational taxonomic units (OTUs) were clustered using USEARCH and taxonomically classified with the RDP classifier in 16S modus (confidence value > 0.8). Finally, obtained raw reads were mapped back onto the OTU sequences to get quantitative assignments. Normalized calculation of the Shannon index and the Simpson index was performed by an inhouse perl script.

### In situ quantitation of mucosal T cells

Immunostaining on paraffin sections was performed as previously described^1–3,42^. The primary antibodies was mouse anti-CD4 (clone 1F6; Novocastra, Newcastle, UK). For detection, the streptavidin–alkaline phosphatase kit with Fast Red as chromogen (Dako) was used according to the manufacturer’s instructions. Nuclei were counterstained with hematoxylin (Merck, Darmstadt, Germany). Positive cells within the lamina propria were quantified in colonic tissues per high-power field (hpf; 0.237mm^2^), and 10 hpf were averaged in each case^1–3,42^.

### Mucosal cell isolation and flow cytometric analysis

Mucosal mononuclear cells were isolated from colonic biopsy specimens by collagenase type II (Sigma, Hamburg, Germany) digestion as described previously^2,3^. The following antibodies were used for phenotypic analysis: anti-CD3-allophycocyanin (APC), anti-CD3-peridinin chlorophyll protein Cy5.5 (PerCPCy5.5; both clone SP34; BD Biosciences), anti-CD4-PerCPCy5.5, anti-CD4-APC (both L200; BD), anti-CD8-phycoerythrin (PE), anti-CD8-fluorescein (FITC; both DK25; Dako), anti-CD45RA-FITC (ALB11; Beckman Coulter), and anti-CCR5-PE (3A9; BD). Lymphocytes were gated on the basis of characteristic forward and side scatter properties. CD4^+^ T cells were identified by co-expression of CD3 and lack of CD8 expression and memory CD4^+^ T cells were identified by lack of CD45RA expression on CD4^+^ T cells. The total number of analyzed colonic cells was between 360,000 and 550,000 and cells in the CD3^+^ T cell gate consisted of 14,000 – 44,000 colonic cells. CD4^+^ T cell counts in fresh whole blood were quantified by the use of Trucount Tubes (BD) according to the manufacturer’s instructions. Data were acquired on the FACS Calibur (BD) and analyzed with FlowJo software version 8.8.4. (BD).

### Short-term colonic tissue culture and quantitation of mucosal cytokine secretion and microbial products

Short-term culture supernatants of colonic biopsy specimens were prepared as described before^59^. Briefly, biopsy specimens were washed, weighted, and incubated in 200 µL of fetal calf serum-free Roswell Park Memorial Institute 1640 medium (Gibco-BRL) containing 100 U/mL penicillin, 100 µg/mL streptomycin, and 2.5 µg/mL amphotericin (Seromed Biochrom KG) for 48 h at 37 °C in a humidified 5% CO_2_/70% O_2_ air atmosphere. Decay of bacteria with release of bacterial components was achieved by culture media supplemented with antibiotics. The contents of LPS, acetate, and butyrate were quantified with Enzyme-linked Immunosorbent Assays for LPS or Butyric Acid (both Cloud-Clone Corp., USA) and an Acetate Colorimetric Assay (Sigma Aldrich, USA) according to the manufacturer’s protocols. IL-2 and IL-4 were quantified in the supernatants with cytometric bead arrays (BD) according to the manufacturer’s protocol.

### Quantitation of biomarkers of enterocyte damage and microbial translocation

Commercially available assay kits were used according to manufacturer’s protocols to quantify I-FABP and LBP (both Hycult Biotech, Uden, The Netherlands) in serum.

### Statistical analysis

Data were analyzed using the paired t-test, if they passed the Kolmogorov–Smirnov normality test or the Wilcoxon matched-pairs signed rank test. Correlations and statistical significance were determined by the Spearman rank correlation test. All data were statistically analyzed with Prism software version 5.0 (Graph Pad Inc., La Jolla, CA).

## Data Availability

The sequence data generated and analysed during the current study are available in the EMBL-EBI SRA repository under the Bioproject ID PRJEB31033.
